# Flying Small Target Detection for Anti-UAV Based on a Gaussian Mixture Model in a Compressive Sensing Domain

**DOI:** 10.3390/s19092168

**Published:** 2019-05-10

**Authors:** Chuanyun Wang, Tian Wang, Ershen Wang, Enyan Sun, Zhen Luo

**Affiliations:** 1 School of Computer Science, Shenyang Aerospace University, Shenyang 110136, China; sunenyan@sau.edu.cn (E.S.); luozhen@sau.edu.cn (Z.L.); 2 School of Automation Science and Electrical Engineering, Beihang University, Beijing 100191, China; wangtian@buaa.edu.cn; 3 School of Electronic and Information Engineering, Shenyang Aerospace University, Shenyang 110136, China; wes2016@sau.edu.cn

**Keywords:** flying small target detection, anti-UAV, gaussian mixture model, compressive sensing, low-rank and sparse matrix decomposition

## Abstract

Addressing the problems of visual surveillance for anti-UAV, a new flying small target detection method is proposed based on Gaussian mixture background modeling in a compressive sensing domain and low-rank and sparse matrix decomposition of local image. First of all, images captured by stationary visual sensors are broken into patches and the candidate patches which perhaps contain targets are identified by using a Gaussian mixture background model in a compressive sensing domain. Subsequently, the candidate patches within a finite time period are separated into background images and target images by low-rank and sparse matrix decomposition. Finally, flying small target detection is achieved over separated target images by threshold segmentation. The experiment results using visible and infrared image sequences of flying UAV demonstrate that the proposed methods have effective detection performance and outperform the baseline methods in precision and recall evaluation.

## 1. Introduction

In recent years, the Unmanned Aerial Vehicles (UAV) industry has been booming at a rate and scale and has gained emerging interest in both civilian and military applications. Especially, the consumer micro-UAV, such as drones, is growing exponentially and joins in the airspace setting for commercial and individual needs, such as aviation videography, courier services, traffic surveillance and land surveying [1]. However, as the rapid spread of micro-UAV, many new security threats and social problems, including disturbing aviation safety of commercial aircraft, breaching no-fly security in sensitive areas and invading public privacy, frequently occur and appear to be continually  increasing [2].

In spite of the laws and regulations being enforced and no-fly zone control being built in micro-UAV to restrain operators’ behavior, there still exists potentially dangers due to the unauthorized flying with spontaneous. As a consequence, there is a critical need for an anti-UAV system to detect a micro-UAV that enters a specific sensitive airspace in time. The traditional technology of anti-UAV is radar which works by means of active illumination with electromagnetic waves [3]. In the low-attitude airspace context, the radar signals of targets immerse in complex background clutter, e.g., trees, buildings [4,5]. Moreover, potentially dangerous targets should be detected when they are still far away, which means they may still be very small. Thereupon, the cost performance of expensive radar for micro-UAV detection is greatly reduced.

Fortunately, vision sensors are low-cost, passive and robust to jamming; hence, motivating their use for micro-UAV detection, even though they have a limited range compared to radars. As a matter of fact, there still exists many challenges to detect a micro-UAV with a visual method. This is because it not only occupies a small portion of the field of view from a far distance and flies against complex backgrounds under various weather and lighting conditions, but also appears changing size and diverse shapes in fully 3D dimensional environment.

Over the past few years, the problems of anti-UAV have been attracting more and more attention, but only a few studies of visual detection are studied. A non-technical way of anti-UAV was gathering information about potentially illegal or dangerous activity of UAV via crowd sourcing from their mobile phone by submitting a short online DroneALERT incident report [6]. The drone surveillance technologies and the existing anti-drone systems are comprehensively reviewed [2], and an anti-drone system called ADS-ZJU is developed by utilizing audio, video and RF technologies [7]. Sosnowski et al. presented a concept of short-range air defence systems to protect against drone threats with various optoelectronic sensors [8]. Inspired by different optical properties possessed by small drones with the sky as a background, a combination of infrared polarization filters was suggested to identify drones [9]. In addition, multiple local features of aircraft was used to compose a feature clustering criterion for vision based aircraft tracking [3]. Furthermore, some researchers have attempted to detect and track flying small UAVs from a single moving camera. Li et al. classified candidate targets by using their spatio-temporal traits on the basis of background motions estimation and distinctive points identification, and then a Kalman filter was used to boost the tracking performance [10]. Rozantsev et al. achieved object-centric motion stabilization of image patches by a regression-based approach, and  then detected flying objects by combining both appearance and motion cues as spatio-temporal image cubes [11,12]. In addition to these, the research of vision-based sense-and-avoid for micro-UAV could provide a reference [13,14].

In this study, multiple visual sensors are distributed around the surveillance area, and no-blind area coverage for surveillance is realized in a cooperative manner, so as to build an intelligent visual surveillance system for discovering the approaching UAV in time [15,16]. However, due to the optical properties and image resolution of visual sensors, it is limited to detect a target as far as possible. In  that case, the size of a target is often very small and with only a few pixels because of a long imaging distance, as well as the energy of the target received by an imaging sensor is very low and with dim spotlike features because of atmospheric effects [17,18,19]. Therefore, an efficient flying small target detection algorithm is crucial to give a warning as early as possible.

In this paper, we concentrate on flying small target detection for anti-UAV from a stationary camera. Making use of the advantages of compressive sensing which can efficiently compress a higher-dimensional data to lower-dimensional one and perfectly reconstruct from a small number of linear measurements with high probability, the image background modeling for flying small target detection is no longer over each pixel but over each linear measurement. Subsequently, the images in sequence are converted into patches to construct a background model for better enforcing the image neighboring correlation, and then the candidate patches which perhaps contain targets that are identified by using background models of each patch. Moreover, with the characteristics of background scene slight change and target relative salient movement within a finite time period, a matrix composed of images in sequence is separated into background images and target images by low-rank and sparse matrix decomposition. As a Consequence, flying small target detection for anti-UAV is achieved over a separated target image.

The remainder of this paper is organized as follows. Firstly, a new Gaussian mixture model in a compressive sensing domain is designed in Section 2. Section 3 presents the approach to flying small target detection composed by candidate patches identification, local foreground-background images separation and comprehensive scheme of the proposed method. Section 4 contains experiments to demonstrate the effectiveness of the proposed method and compare its performance with baseline methods. Finally, conclusions are drawn in Section 5.

In order to clearly depict this work, the major notations used throughout the rest of this paper are briefly summarized in Table 1.

## 2. Gaussian Mixture Modeling in a Compressive Sensing Domain

Traditionally, background modeling has been attracting significant attention in recent years for target detection in the foreground. To tackle this problem, the Gaussian mixture model (GMM) is proposed as a statistical approach by training multiple models of probability density functions over each pixel [20,21]. If a variable *x* is Gaussian distribution with mean μ and variance $\sigma^{2}$, its probability density function (PDF) is defined as

(1) $$N\left(x;\mu ,\sigma^{2}\right)=\frac{1}{\sqrt{2\pi \sigma^{2}}}e^{-\frac{{\left(x-\mu \right)}^{2}}{2\sigma^{2}}}$$

However, the gaussian distribution is a single modal distribution, which can not provide a good approximation for the data distribution of multimodal states. Fortunately, according to previous literatures, GMM is considered to fit any probability distribution of a variable, that is to say, any variable can be regarded as with probability of multiple specific gaussian distributions [22]. Consequently, the GMM is composed by multiple Gaussian distributions, and its PDF is written as the linear sum of PDFs of the Gaussian distributions

(2) $$p\left(x\right)=\sum_{i=1}^{s}p(i)p(x|i)=\sum_{i=1}^{s}w_{i}N\left(x;\mu_{i},\sigma_{i}^{2}\right)$$

where *s* is the number of Gaussian distribution, $w_{i}$, $\mu_{i}$ and $\sigma_{i}^{2}$ are the weight, mean and variance of the ith Gaussian distribution, respectively. In general, *s* is set to 3∼5, and the Gaussian distributions are independent to each other [23,24]. In this study, the flying target with salient movement is the foreground, while the background scene changes are relatively slight. Thereupon, a  Gaussian distribution will catch the pattern of follow-up compressive sensing measurements.

The pixel-based background modeling algorithms establish models for each pixel of an image and classify a new pixel as foreground if its PDF value is below a threshold [25]. Within this general paradigm, these algorithms require high computational complexity and energy consumption as well as large storage. As a new sampling theory, compressive sensing (CS) breaks through the Shannon/Nyquist sampling theorem which states that the sampling rate must be at least twice the highest frequency [26]. Compressive sensing is able to collect and keep sufficient information of a given signal by a small number of linear measurements. Correspondingly, perfect reconstruction or robust approximation of signal relies on effective sparse signal representation and appropriate recovery algorithms [27]. Therefore, background modeling can be performed on these linear measurements, as they are a low-dimensional compressive representations of the given signal.

An image is stacked to form a vector $x={\left[x_{1},x_{2},\cdots ,x_{n}\right]}^{T}\in R^{n}$. The linear projection of the image vector on the measurement matrix $\Phi \in R^{m\times n}\left(m\ll n\right)$ is defined as

(3) y=Φx

where $y={\left[y_{1},y_{2},\cdots ,y_{m}\right]}^{T}\in R^{m}$ represents the low dimensional CS measurement vector.

However, image itself may not be sparse but can be sparsely represented in some transform domain, such as DCT, Fourier, wavelet and over-complete dictionary. Assume that $x\in R^{n}$ can be sparsely represented in terms of a basis of vectors ${\left\{\psi_{i}\right\}}_{i=1}^{q}$ with $\psi_{i}\in R^{n}$, then forming the sparse basis matrix $\Psi =\left[\psi_{1},\psi_{2},\cdots ,\psi_{q}\right]\in R^{n\times q}$ by stacking the vectors as columns, so x can express as

(4) $$x=\sum_{i=1}^{q}\psi_{i}\theta_{i}=\Psi \Theta$$

where $\Theta ={\left[\theta_{1},\theta_{2},\cdots ,\theta_{q}\right]}^{T}\in R^{q}$ is the weighting coefficients vector of x in the Ψ domain [28].

Based on the above discussion, compressive sensing of an image can be described as linear projection process [29]

(5) y=ΦΨΘ=AΘ

where A:=ΦΨ is referred to as sensing matrix. In order for the well-conditioned reconstruction of a *K*-sparse signal x in the specific transform domain, the sensing matrix $A\in R^{m\times q}$ should satisfies the restricted isometry property (RIP) with order 2K [30].

**Definition** **1.** *A matrix A satisfies the restricted isometry property (RIP) of order K if there exists a $\delta_{K}\in \left(0,1\right)$ such that*

$$\left(1-\delta_{K}\right){∥\Theta ∥}_{2}^{2}\leq {∥A\Theta ∥}_{2}^{2}\leq \left(1+\delta_{K}\right){∥\Theta ∥}_{2}^{2},$$

*for all $\Theta \in \{\Theta :∥\Theta {∥}_{0}\leq K\}$, where ${∥\Theta ∥}_{0}:=♯\left(i|\theta_{i}\neq 0\right)$ is the zero pseudo-norm [31].*

Unfortunately, the task of holding that the sensing matrix satisfies the RIP is an NP-hard problem, but an alternative approach is to make sure that the measurement matrix Φ is incoherent with the sparse basis matrix Ψ [29]. The greater incoherence of the measurement/sparsity pair (Φ,Ψ), the smaller number of the linear measurements needed. In a Gaussian random matrix, the elements are independent and identically distributed (i.i.d.) random variables from a zero-mean, 1/n-variance Gaussian density. Then, a Gaussian random matrix is incoherent with most of orthogonal sparse basis matrix, and is chosen as measurement matrix of compressive sensing to collect information of random projections

(6) $$\Phi ={\left[\phi_{1},\phi_{2},\cdots ,\phi_{m}\right]}^{T}$$

where $\phi_{i}\in R^{n}$, and $\phi_{i}$ is Gaussian distribution with *n*-dimension N(0,I).

With these considerations in mind, we replace the pixels with a small set of CS measurements to establish a background model for flying small target detection [32], employing the low dimensional CS measurement vector $y={\left[y_{1},y_{2},\cdots ,y_{m}\right]}^{T}$ instead of the high dimensional image vector $x={\left[x_{1},x_{2},\cdots ,x_{n}\right]}^{T}$ to establish Gaussian mixture model by Equation (2), and this process is so-called Gaussian mixture modeling in a compressive sensing domain (GMM_CS) [33].

When a new image is captured at time *t* during model training, a series of linear projections with Gaussian random matrix is operated over the image vector by Equation (3). Subsequently, each  element in CS measurement vector y is identified with previous Gaussian mixture model by

(7) $$|y_{k}^{t}-\mu_{k,i}^{t-1}|<\xi \sigma_{k,i}^{t-1}\;\left(i=1,2,\ldots ,s\right)$$

where ξ is a constant. If there exists a matched Gaussian distribution, the corresponding mean and variance should be updated by utilizing current CS measurement

(8) $$\mu_{k}^{t}=\left(1-\beta \right)\mu_{k}^{t-1}+\beta y_{k}^{t}$$

(9) $${\left(\sigma_{k}^{t}\right)}^{2}=\left(1-\beta \right){\left(\sigma_{k}^{t-1}\right)}^{2}+\beta {\left(y_{k}^{t}-\mu_{k}^{t}\right)}^{2}$$

where β∈(0,1) represents the learning rate of mean and variance of the Gaussian distribution. Then, the weights of *s* Gaussian distributions are updated simultaneously

(10) $$w_{k,i}^{t}=\left(1-{\left(-1\right)}^{\tau }\alpha \right)w_{k,i}^{t-1}$$

where α∈(0,1) denotes the learning rate of weight of the Gaussian distribution, τ∈{0,1} will set to 1 for the matched Gaussian distribution and to 0 for mismatched one. If there no matched Gaussian distribution, the mean and variance of the Gaussian distribution with minimum weight will be updated, and then the weights of *s* Gaussian distributions are normalized and let $\sum_{i=1}^{s}w_{k,i}=1$.

## 3. Approach to Flying Small Target Detection by Local Image Separation

### 3.1. Candidate Patches Identification Based on GMM_CS

From a general viewpoint, images captured by imaging systems for fly small target detection have large sizes, thus, when we treat a two-dimensional image by vectorizing it into a one-dimensional vector, the corresponding vector is with high dimensions [34]. As a consequence, there are two negative influences. On one hand, a fairly large measurement matrix is needed for CS which makes the storage and computations of a Gaussian ensembly very difficult [35]. On the other hand, the linear projection process over the high dimensional vector of whole image reckons without the image neighboring correlation.

In addressing the problems above, we break the whole image into overlapping or non-overlapping patches by patch transform [36]. Assume a new image is represented by a matrix D, the non-overlapping patch transform will convert the matrix into several block matrices with *r* rows and *c* columns

(11) $$D=\left[\begin{array}{cccc} D_{1,1} & D_{1,2} & \cdots & D_{1,c}\\ D_{2,1} & D_{2,2} & \cdots & D_{2,c}\\ \vdots & \vdots & \vdots & \vdots \\ D_{r,1} & D_{r,2} & \cdots & D_{r,c} \end{array}\right]$$

where $D_{i,j}$ denotes the $i^{th}$ row, $j^{th}$ column image patch represented by a block matrix. For simplicity, each train image and test image are divided into non-overlapping patches for experiment in this study. In spite of the small size of the image patch leading to low computational complexity for one image patch, more image patches which perhaps maintain a flying small target, and subsequently, high computational complexity will be induced in the following process of target detection. On account of these considerations, we designate the size of image patch to refer to the roughly maximum size of flying target and division into integer patches.

Subsequently, the background modeling and target detection procedures will be applied over image patches. The background modeling is to establish a Gaussian mixture model for each linear measurement of image patches generated from a group of training images. After that, the obtained background model based on GMM_CS can be employed to identify the candidate patches which perhaps maintain a flying small target. Thereinto, the identification of an image patch maintains a flying small target, operates in a compressive sensing domain, and includes two steps. The first step is the comparison between each linear measurement of input image patch with the obtained background model by Equation (7); the second step is to compute the statistical ratio of matched linear measurements to the total linear measurements, which is expressed as

(12) $$\delta =\frac{1}{m}\sum_{k=1}^{m}J_{k}$$

where δ∈(0,1] is regard as a threshold for identifying current image patch is maintain a flying small target or not, *m* is the total linear measurements of current image patch, and *J* is defined as

(13) $$J_{k}=\left\{\begin{array}{cc} 1, & \mathrm{if}\;|y_{k}-\mu_{k,i}|<\xi \sigma_{k,i}\\ 0, & \mathrm{otherwise} \end{array}\right.\;\left(i=1,2,\ldots ,s\right)$$

**Figure 1:**
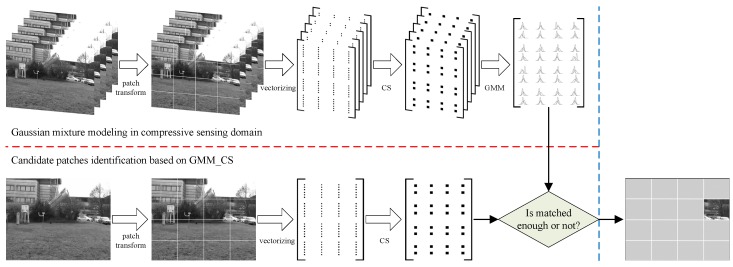
A demonstration of candidate patches identification based on GMM_CS.

Based on the aforementioned discussion, a demonstration of candidate patches identification based on GMM_CS is depicted in Figure 1.

### 3.2. Local Target-Background Patches Separation

**Figure 2:**
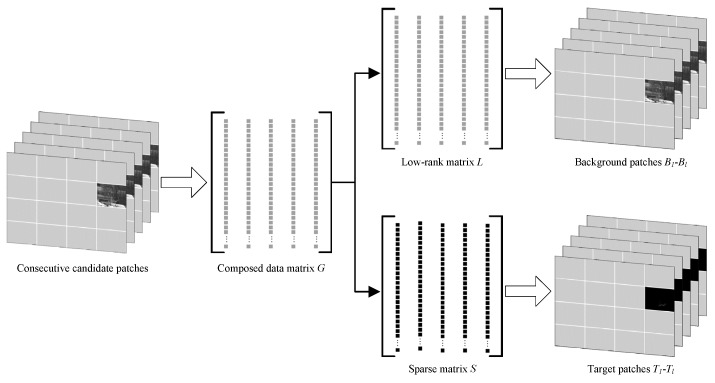
Local foreground-background images separation via RPCA.

Along with continuously captured scene images of the imaging system, a group of consecutive candidate patches extracted form several adjacent images is designated and vectorized as columns to compose a data matrix $G\in R^{d\times l}$, where *d* is the total number of pixels in each candidate patch, and *l* is the number of adjacent images. Making use of the characteristics of background scene slight change and target relative salient movement within a finite time period, the data matrix *G* will be decomposed to a low-rank matrix *L* and a sparse matrix *S* via robust principal component analysis (RPCA) [37,38], where the low-rank matrix *L* can be regard as a composition of the separated background patches $B_{1}-B_{l}$, and the sparse matrix *S* can be regard as a composition of the separated target patches $T_{1}-T_{l}$, as shown in Figure 2.

Compared with the conventional principal component analysis which can offer low-rank representation of a given data matrix corrupted by small i.i.d. Gaussian noise, the RPCA can work well with respect to grossly corrupted observations, and then the low-rank and sparse matrix decomposition can be achieved by many kinds of optimization techniques [39], in which the optimization problem can be formulated as following

(14) $$\min_{L,S}{(\mathrm{rank}\left(L\right)+\lambda ∥\mathrm{vec}\left(S\right)∥}_{0})\;\mathrm{subject}\;\mathrm{to}\;G=L+S$$

where rank(·) is a function to solve the rank of matrix, vec(·) is vectorization for a matrix, ${∥\cdot ∥}_{0}$ is the $\ell_{0}$-norm for vector which denotes the number of non-zero entries, and λ is a weight parameter for tradeoff the low-rank matrix *L* and sparse matrix *S*.

Conventionally, the optimization problem Equation (14) can be exactly solved via convex optimization, in which the rank function is relaxed to the nuclear norm ${∥\cdot ∥}_{*}$ as its convex envelope, and the $\ell_{0}$-norm is relaxed to the $\ell_{1}$-norm as its convex envelope under a certain condition. Consequently, the optimization problem is converted to minimize a combination of the nuclear norm and the $\ell_{1}$-norm [40].

In this study, considering that each column of the data matrix *G* is the vectorized image and the images are obtained from a static camera observing a fixed scene, the rank of the expected low-rank matrix *L* can be approximately estimated. On the other hand, the pixel number of foreground target is specific in a short time, so the sparseness of the expected sparse matrix *S* is also appreciable. Therefore, the optimization problem Equation (14) can be recast into a combination of the rank-*R* matrix and *K*-sparse matrix approximation problem

(15) $$\min_{L,S}{∥\mathrm{vec}\left(G-\left(L+S\right)\right)∥}_{2}\;\mathrm{subject}\;\mathrm{to}\;\mathrm{rank}\left(L\right)\leq R,\;{∥\mathrm{vec}\left(S\right)∥}_{0}\leq K$$

where ${∥\cdot ∥}_{2}$ is the $\ell_{2}$-norm for vector.

To solve the NP-hard problem, a  pursuits algorithm dubbed SpaRCS for sparse and low-rank decomposition via compressive sensing is adopted [41]. Note that, in this study, the linear operator $A:R^{d\times l}\to R^{N}$ is vectorization of a matrix, and its adjoining operator $A^{*}:R^{N}\to R^{d\times l}$ denotes rearrangement from a vector to a matrix. The low-rank matrix *L* and the sparse matrix *S* are iteratively estimated by constructing two bases and attempting matrix recovery restricted to the bases. At each iteration, five steps: supports identification, supports merging, least squares estimation, supports pruning and residue updating are respectively processed to update its estimates of *L* and *S*.

On one hand, the low-rank matrix *L* is approximated by a set of up to *R* rank-1 matrices arranged from its singular vectors, and the set of entire singular vectors is defined as

(16) $$O=\{\left(u_{i},v_{i}\right):\left(U,\Sigma ,V\right)=\mathrm{svd}\left(\cdot \right)\}$$

where $u_{i}$ and $v_{i}$ are respectively the $i^{th}$ column vector of matrices *U* and *V*, and svd(·) is a function to singular value decomposition (SVD) of a given matrix. Further assume that a subset of singular vectors Ω⊂O, and let $F_{\Omega }\left(\cdot \right)$ denotes the projection operator onto the subspace spanned by Ω, then  the estimation of low-rank matrix can be converted to a optimization problem about Frobenius norm of the projection matrix [42].

On the other hand, the sparse matrix *S* is approximated by the largest *K*-term support set of the given matrix, and the set of total practicable terms is defined as

(17) Q={(i,j):i=1,2,…d;j=1,2,…l}

where (i,j) denotes the position of support term. Further assume that a subset of support terms Ω⊂Q, then let $H_{\Omega }\in R^{d\times l}$ denotes a 0-1 matrix with entity

(18) $$h_{ij}=\left\{\begin{array}{cc} 1, & \mathrm{if}\;(i,j)\in \Omega \\ 0, & \mathrm{otherwise} \end{array}\right.\;\left(i=1,2,\ldots d;j=1,2,\ldots l\right)$$

Subsequently, the estimation of sparse matrix can be converted to a optimization problem about Frobenius norm of dot product matrix [43].

### 3.3. Comprehensive Scheme and Algorithm Implementation

**Figure 3:**
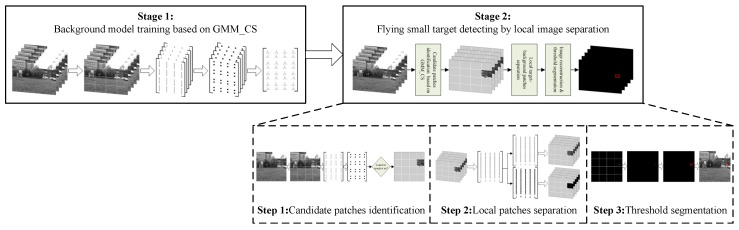
Comprehensive scheme for flying small target detection.

Based on the aforementioned discussion, a new approach to flying small target detection for anti-UAV based on a Gaussian mixture model in a compressive sensing domain is designed. The  approach includes two operation stages: background model training and flying small target detecting, in which the flying small target detecting stage is composed by three process parts: candidate patches identification, local foreground–background image separation and threshold segmentation over reconstructed sparse images. The comprehensive scheme of the proposed approach is depicted in Figure 3.

Correspondingly, the synthetic integrated detection approach to flying small target in image sequence can be presented based on all above discussed modules, and outlined as Algorithm 1.

**Algorithm 1** Approach to flying small target detection based on GMM_CS.**Input:** Flying small target image sequence**Output:** Detection results of each image in sequence
  1:Initialize $N_{train}$, $N_{detect}$, *s*, *m*, ξ, α, β, *R*, *K*, ϵ  2:
**for** $i=1\;\mathrm{to}\;N_{train}$ **do**
  3: Break image into patches by Equation (11) and compressive sensing by Equation (3)  4: Make GMM_CS for each patch by Equations (7)–(10)  5:
**end for**
  6:
**for** $i=1\;\mathrm{to}\;N_{detect}$ **do**
  7: Break image into patches by Equation (11) and compressive sensing by Equation (3)  8: Identify candidate patches by Equations (7), (12), (13)  9: **for** $j=1\;\mathrm{to}\;N_{patch}$ **do** 10:  Designate successive patches and compose a data matrix *G* 11:  **repeat** 12:   Set k=0,${\hat{L}}_{0}=0$,${\hat{S}}_{0}=0$,${\hat{\Omega }}_{L}=\left\{\right\}$,${\hat{\Omega }}_{S}=\left\{\right\}$,$P_{0}=G$ 13:   Update indicator k=k+1 14:   Identify supports: $\Omega_{L}^{\prime }=\arg\max_{\Omega \subset O}\left\{∥F_{\Omega }\left(P_{k-1}\right){∥}_{F}:|\Omega |\leq 2R\right\}$ 15:   Merging: ${\tilde{\Omega }}_{L}={\hat{\Omega }}_{L}\cup \Omega_{L}^{\prime }$ 16:   Estimating: ${\tilde{L}}_{k}=\arg\min_{L}\left\{∥G-L-{\hat{S}}_{k-1}{∥}_{F}:L\in \mathrm{span}\left({\tilde{\Omega }}_{L}\right)\right\}$ 17:   Pruning: ${\hat{\Omega }}_{L}=\arg\max_{\Omega \subset O}\left\{∥F_{\Omega }\left({\tilde{L}}_{k}\right){∥}_{F}:|\Omega |\leq R\right\}$, ${\hat{L}}_{k}=F_{{\hat{\Omega }}_{L}}\left({\tilde{L}}_{k}\right)$ 18:   Update residue: ${\tilde{P}}_{k}=G-{\hat{L}}_{k}-{\hat{S}}_{k-1}$ 19:   Identify supports: $\Omega_{S}^{\prime }=\arg\max_{\Omega \subset Q}\left\{∥H_{\Omega }\cdot {\tilde{P}}_{k}{∥}_{F}:|\Omega |\leq 2K\right\}$ 20:   Merging: ${\tilde{\Omega }}_{S}={\hat{\Omega }}_{S}\cup \Omega_{S}^{\prime }$ 21:   Estimating: ${\tilde{S}}_{k}=\arg\min_{S}\left\{∥G-{\hat{L}}_{k}-S{∥}_{F}:S=H_{\Omega }\cdot {\tilde{P}}_{k},\Omega \subset {\tilde{\Omega }}_{S}\right\}$ 22:   Pruning: ${\hat{\Omega }}_{S}=\arg\max_{\Omega \subset Q}\left\{∥H_{\Omega }\cdot {\tilde{S}}_{k}{∥}_{F}:|\Omega |\leq K\right\}$, ${\hat{S}}_{k}=H_{{\hat{\Omega }}_{S}}\cdot {\tilde{S}}_{k}$ 23:   Update residue: $P_{k}=G-{\hat{L}}_{k}-{\hat{S}}_{k}$ 24:  **until** $∥G-{\hat{L}}_{k}-{\hat{S}}_{k}{∥}_{F}/{∥G∥}_{F}\leq \epsilon$ 25: **end for** 26: Detect target over reconstructed sparse images by threshold segmentation 27:
**end for**



## 4. Experimental Results and Analysis

### 4.1. Experiment Setting and Evaluation Metrics

In order to validate the proposed detection method of flying small target, two experiments are conducted on visible and infrared image sequences of flying UAVs derived from two challenging datasets. The image sequence for the first experiment has a 189-frame visible image with resolution 640×480 pixels acquired by a CCD visible light camera, selected from the video named ‘video_37.avi’ in the UAV dataset which was collected by Rozantsev et al. [11]. The video with 14 s and 25 frames per seconds is the only one drone video captured from a stationary camera. Due to some discontinuous frames being without a ground-truth, we choose the labeled 189-frame visible image of this video for the experiment. The image sequence for the second experiment has a 337-frame infrared image with resolution 640×480 pixels acquired by a longwave FLIR A655sc camera, selected from the sequence named ‘quadrocopter2’ in the LTIR dataset which was used in the Visual Object Tracking (VOT) challenge [44]. The image sequence is captured from a stationary camera. Due to the moving foreground of flying target not being salient with no obvious changes, we choose 337 frames from the original 1010 frames by a three-frame interval for the experiment.

Additionally, the true positives (TP), false positives (FP) and false negatives (FN) of flying UAVs are counted, and then two most important evaluation metrics: the recall and precision are calculated to quantitatively evaluate the performance of the proposed flying small target detection method, and they are defined as follows:

(19) $$Recall=\frac{TP}{TP+FN}$$

(20) $$Precision=\frac{TP}{TP+FP}$$

In the evaluation, we regarded the overlap score between detected bounding boxes $r_{d}$ and manually labeled ground-truth bounding boxes $r_{t}$ that are greater than a certain threshold as correct detections and boxes that less than a certain threshold as misdetection, and the overlap score is defined as

(21) $$Score=\frac{|r_{d}\cap r_{t}|}{|r_{d}\cup r_{t}|}$$

where ∩ and ∪ represent the intersection and union of two regions, respectively. |·| denotes the number of pixels in the region.

All the experiments are carried out on a general computer with 8-GB memory and 2.2-GHz Intel Core i5-5200U processor.

### 4.2. Performance Evaluation and Comparative Analysis

To test the effectiveness and practicality of the proposed method, its performance is evaluated by the aforementioned metrics and compared with three baseline methods. One is a well-studied background subtraction method based on an improved adaptive Gaussian mixture model (GMMv1) [45], another is a superior foreground and background separation method named Grassmannian robust adaptive subspace tracking algorithm (GRASTA) [46]. the third one is a state-of-the-art moving target detection method by detecting contiguous outliers in the low-rank representation (DECOLOR) [47].

The GMMv1 method is performed in a background subtraction library named BGSLibrary developed by Sobral [48], and its parameter setting in the library is adopted. The GRASTA and DECOLOR methods are implemented as open source and provided in this paper, and 20 frames visible or infrared image are introduced to train the initial subspace of GRASTA method, while the parameters of DECOLOR method employ the default values provided by the author.

The proposed method is implemented and executed on a Matlab R2012a software platform. In the following experiments, the number of Gaussian distribution is s=3. Correspondingly, the learning rate of weight is α=0.2 and the learning rate of mean and variance is β=0.2 for each Gaussian distribution. In the procedure of CS measurement, the compressive ratio is m=0.5n. Moreover, the rank of the expected low-rank matrix *R* is set to 1, and the sparseness of the expected sparse matrix *K* is set to 0.1 times the number of pixels in one image patch. For identifying a image patch with a flying small targets, the threshold of δ is set to 0.25m and 0.75m for the visible and infrared image sequences, respectively. In addition, 20 frames of visible or infrared images are introduced to train the proposed GMM_CS, and all images are broken into non-overlapping patches with a resolution of 80×60 and 64×48 pixels for the visible and infrared image sequences, respectively.

**Figure 4:**
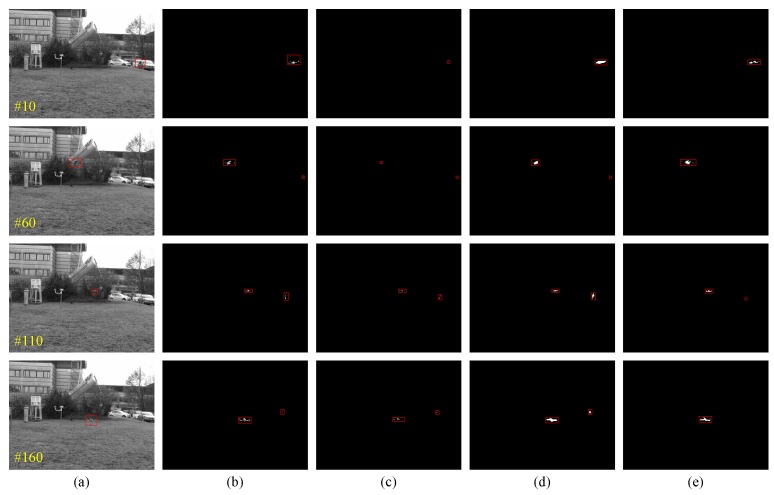
Some experiment results of flying small target detection on visible image sequence obtained from different algorithms (**a**) groundtruth, (**b**) GMMv1, (**c**) GRASTA, (**d**) DECOLOR and (**e**) the proposed method.

In the first experiment, a visible image sequences of a flying drone is tested to detect using three baseline methods and the proposed method. Due to the changing flight attitude, near or far distance and variable lighting conditions, the shape and size of the drone is in flux and is challenging for detection. As an intuitively illustration, the flying small target detection results of four in-between frames are given as shown in Figure 4. The yellow number marked in the lower left quarter of image is the serial number of the image frame, and the target region of groundtruth, detected region by GMMv1, GRASTA, DECOLOR and the proposed method in this study are marked by a red bounding box. These results show that the proposed method can effectively detect the actual flying small target and generate less false alarm targets relative to the baseline methods.

**Figure 5:**
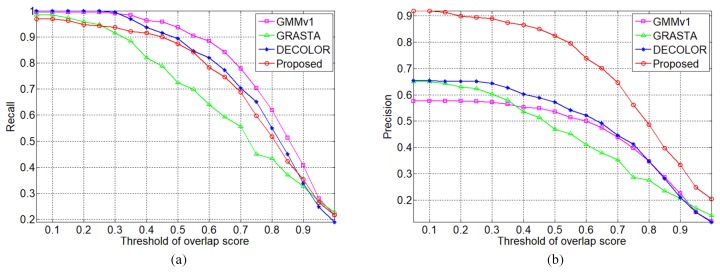
Curves of recall and precision with different threshold of overlap scores for flying small target detection on visible image sequence (**a**) recall and (**b**) precision.

In order to objectively quantitate the evaluation, the recall and precision are statistically calculated over the whole visual small target sequences. The thresholds of overlap score for target success detection affirmation are choose from 0.05 to 1 with step 0.05, and the result curves are plotted in Figure 5. It can be observed visually that the higher threshold of overlap score leads to lower recall and precision. Note that the proposed method outperforms the baseline methods in precision evaluation while they have approximately equal recall evaluation.

**Figure 6:**
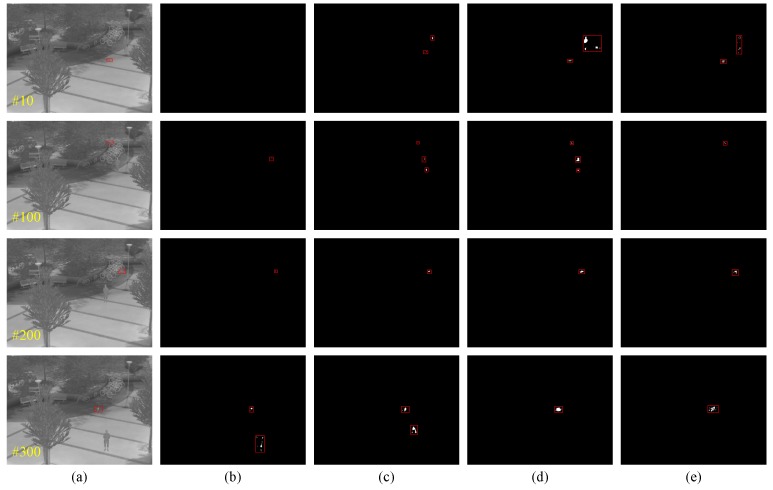
Some experiment results of flying small target detection on infrared image sequence obtained from different algorithms (**a**) groundtruth, (**b**) GMMv1, (**c**) GRASTA, (**d**) DECOLOR and (**e**) the proposed method.

In the second experiment, a longwave infrared image sequences of flying quadrocopter is tested to detect by three baseline methods and the proposed method. Due to the sequence coming from a surveillance scenario with long imaging distance, the flying quadrocopter presents not only small size but also dim intensity even immersing in complex background. so it is rarely perfectly visible by the naked eye, not to mention automatic detection. For clear performance demonstration, the flying small target detection results in four in-between frames that are given as shown in Figure 6. The yellow number marked in the lower left quarter of the image is the serial number of the image frame, and the target region of groundtruth, detected region by GMMv1, GRASTA, DECOLOR and the proposed method in this study are marked by a red bounding box. We can find that the actual flying small target still can be detected in the challenging context in spite of being about some false alarm targets. Especially, the detection performance of the proposed method is not inferior to any baseline methods.

**Figure 7:**
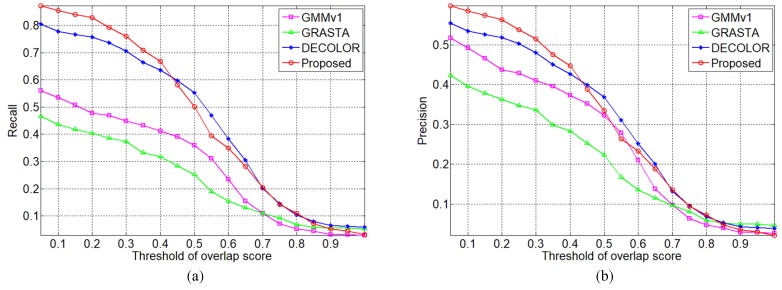
Curves of recall and precision with different threshold of overlap scores for flying small target detection on infrared image sequence (**a**) recall and (**b**) precision.

Consequently, the recall and precision are statistically calculated over the whole infrared small target sequences. The thresholds of overlap score for target success detection affirmation are choose from 0.05 to 1 with step 0.05, and the resultant curves are plotted in Figure 7. It is easy to observed that the proposed method has better recall and precision evaluation when the thresholds of overlap score is less than 0.45, and has similar recall and precision evaluation when the thresholds of overlap score is more than 0.45.

## 5. Conclusions

In this paper, a new Gaussian mixture model in a compressive sensing domain is designed, and then a flying small target detection method for anti-UAV is proposed. The method not only makes use of the advantages of compressive sensing to reduce data quantity of background modeling, but also achieves flying small target detection on a handful of candidate patches by low-rank and sparse matrix decomposition. The results of experiments show that the proposed method can effectively detect flying small targets with less false alarms, and has outstanding recall and precision performance on both visible and infrared image sequences. In future studies, we will aim to focus on distributed deployment of multiple visual sensors for barrier coverage of surveillance, as well as multi-view stereo vision for flying small target detection by multi-sensor cooperation.

## Figures and Tables

**Table 1:** A brief summary of the notations used throughout the rest of this paper.

Notations	Descriptions
*s*	the number of Gaussian distribution
$w_{i}$	the weight of the $i^{th}$ Gaussian distribution
$\mu_{i}$	the mean of the $i^{th}$ Gaussian distribution
$\sigma_{i}^{2}$	the variance of the $i^{th}$ Gaussian distribution
x	a vector stacked by an image with *n* pixels
y	a compressive sensing measurement vector with *m* dimensions
Ψ	a sparse basis matrix composed by *q n*-dim basis vectors
Φ	a measurement matrix composed by *m n*-dim Gaussian distribution
Θ	the weighting coefficients vector of x in the Ψ domain
ξ	a constant for Gaussian distribution identification
α	the learning rate of weight of the Gaussian distribution
β	the learning rate of mean and variance of the Gaussian distribution
τ	the value of the Gaussian distribution matched or not
$J_{k}$	the value of the $k^{th}$ linear measurement matched or not
δ	a threshold for identifying image patch maintain target or not
D	a matrix composed by *r* rows and *c* columns block matrices
*G*	a matrix composed by a group of vectorized consecutive patches
*L*	the low-rank matrix decomposed from matrix *G* via RPCA
*S*	the sparse matrix decomposed from matrix *G* via RPCA
*R*	the rank of the low-rank matrix *L*
*K*	the sparse degree of the sparse matrix *S*
λ	a weight parameter for tradeoff the matrices *L* and *S*
$u_{i}$	the $i^{th}$ column vector of matrix *U* after SVD
$v_{i}$	the $i^{th}$ column vector of matrix *V* after SVD
$\Omega_{L}$	a subset of the entire set of singular vectors O
$\Omega_{S}$	a subset of the entire set of position support terms Q
